# The Home as a Modulator of Milk Immunity: Association Between Domestic Factors and Immune Cell Populations in Human Breast Milk

**DOI:** 10.3390/nu17152574

**Published:** 2025-08-07

**Authors:** Agata Tomaszewska, Klaudia Porębska, Alicja Jeleniewska, Katarzyna Królikowska, Agnieszka Lipińska-Opałka, Agnieszka Gościńska, Robert Zdanowski, Milena Pogonowska, Bolesław Kalicki

**Affiliations:** 1 Department of Pediatrics, Nephrology and Allergology, Military Institute of Medicine—National Research Institute, Szaserów 128, 04-141 Warsaw, Poland; awesolowska@wim.mil.pl (A.J.); kwisniewska1@wim.mil.pl (K.K.); alipinska@wim.mil.pl (A.L.-O.); agoscinska@wim.mil.pl (A.G.); mpogonowska@wim.mil.pl (M.P.); kalicki@wim.mil.pl (B.K.); 2 Faculty of Medicine, University of Warsaw, Krakowskie Przedmieście 26/28, 00-927 Warsaw, Poland; 3 Laboratory of Molecular Oncology and Innovative Therapies, Military Institute of Medicine—National Research Institute, Szaserów 128, 04-141 Warsaw, Poland; kporebska@wim.mil.pl (K.P.); rzdanowski@wim.mil.pl (R.Z.)

**Keywords:** breast milk, milk composition, domestic environment, co-sleeping, siblings, parity, pets

## Abstract

**Background/Objectives:** Human breast milk is a biologically active fluid. It contains immune cells, stem cells, epithelial cells, and lactocytes. These components may support infant development and immune defense. While milk composition is known to vary with physiological and nutritional factors, the impact of the home environment remains poorly understood. The aim of this study was to examine how selected conditions affect the cellular composition of breast milk. **Methods:** We conducted a cross-sectional study involving 49 lactating mothers of healthy infants under 6 months of age. Breast milk samples were analyzed using flow cytometry. We measured proportions of immune cells (CD3^+^, CD4^+^, CD8^+^, CD19^+^, and CD16/56^+^), hematopoietic stem cells (CD34^+^), mesenchymal stem cells (CD105^+^, CD73^+^, and CD44^+^), and lactocytes (CD326^+^ CD73^+^ and CD326^+^ CD73^−^ phenotypes). Participants completed a questionnaire assessing number of children, co-sleeping, pet ownership, and number of household members. **Results:** Mothers with more than one child showed higher percentages of CD4^+^ (*p* = 0.047) and CD8^+^ (*p* = 0.031) T cells and fewer CD73^+^ lactocytes (*p* = 0.028). Co-sleeping was associated with lower levels of CD3^+^ T cells in milk (*p* = 0.021). Pet ownership correlated with a lower proportion of cytotoxic CD8^+^ cells (*p* = 0.048). The number of household members had no significant effect. **Conclusions:** Domestic factors such as number of children, co-sleeping, and pet exposure are associated with shifts in the immune and lactocyte cell composition of breast milk. These findings suggest that breast milk dynamically adapts to maternal and household-level immune stimuli.

## 1. Introduction

Human breast milk is not only a source of nutrients but also a unique biological substance. It plays a key role in shaping the infant’s immune system [1]. It contains numerous bioactive components, including antibodies, oligosaccharides, cytokines, and enzymes [2,3,4]. In parallel, human milk oligosaccharides (HMOs)—now widely incorporated into infant formulas—have been demonstrated to modulate immune function, support gut barrier integrity, and shape infant microbial colonization [5].

Particular attention has been given to the immune cell content of milk. Recent studies confirm the presence of leukocytes [6], progenitor cells [7], and mesenchymal stem cells [8,9,10]. A distinct and notable cell population is that of lactocytes. These are secretory cells derived from the inner epithelial layer during pregnancy and lactation [11]. They are the predominant cell type in human milk, but their function and characteristics remain insufficiently understood [12].

The role of some immune cells in milk is well established. Maternal leukocytes in breast milk contribute to the infant’s active immune defense by recognizing and neutralizing pathogens [13]. The function of stem cells in milk is still under investigation. In vitro studies show that they can differentiate into adipocytes, chondrocytes, osteoblasts, neurons, hepatocyte-like cells, and pancreatic beta cells. These cells may support tissue proliferation, development, and epigenetic regulation in the infant [9]. Experimental data suggest that milk-derived stem cells can cross the infant’s gastrointestinal barrier, enter circulation, and migrate to organs, where they differentiate into functional cells [14].

The cellular composition of breast milk is dynamic. It is influenced by multiple factors, including stage of lactation [15], infections in the mother or child [9], maternal lifestyle [16], diet [17,18,19], and even time of day [20,21]. Some evidence also suggests that cytokine levels and immune cell counts in milk respond to local and systemic inflammation [22].

Despite growing interest in factors shaping milk composition, little is known about how the home environment affects milk cell populations. Environmental studies have focused mainly on prenatal and early postnatal exposures and their effects on the child’s immune system. It remains unclear whether these exposures influence the immune properties of milk itself.

Domestic factors such as co-sleeping, number of household members, number of children, and pet ownership have not been studied in this context. These variables are known to shape environmental microbiota and immune exposure and may thus influence the cellular composition of milk [23,24,25]. One possible mechanism is through modulation of maternal immune activation. Environmental exposure to endotoxins, allergens, and microbes can affect recruitment of immune cells and the function of mammary epithelial cells [26].

Based on these observations, we hypothesize that the cellular composition of breast milk is associated with selected aspects of the home environment. Although several studies have described the immune composition of milk, no previous study has explored how this composition varies with domestic conditions.

The aim of this study was to investigate the relationship between selected environmental factors and milk cellular composition. We analyzed the effects of number of children, co-sleeping, number of household members, and pet ownership on the abundance of key milk cell populations.

## 2. Materials and Methods

### 2.1. Study Design

This cross-sectional study was conducted between May 2023 and May 2024.

### 2.2. Study Population

Forty-nine breastfeeding mothers were enrolled. All participants were breastfeeding mothers of healthy infants under six months of age. Participants were recruited from the general population of Warsaw using multiple channels. Information regarding this study was posted on the official hospital website and distributed via leaflets in prenatal education centers, especially birthing schools. Additionally, invitations were shared on blogs and online forums dedicated to breastfeeding.

All participants were screened according to predefined inclusion and exclusion criteria. Eligible mothers had term infants (≥38 weeks’ gestation) with appropriate birth weight (between the 10th and 90th percentile). Exclusion criteria included current respiratory infections in the mother or infant, chronic illnesses in either, or a history of mastitis.

At enrollment, all infants were exclusively or predominantly breastfed and had not received solid foods. Table 1 presents the inclusion and exclusion criteria.

### 2.3. Data Collection

Each participant underwent a medical interview and physical examination (mother and child). The interview covered maternal age, infant sex, birth weight, Apgar score, formula supplementation, mode of delivery, and gestational age.

Environmental and lifestyle data were collected via a custom questionnaire developed for this study. The questionnaire was completed independently by participants after providing informed consent. It included questions on the number of household members, presence of siblings, pet ownership, and co-sleeping practices. Co-sleeping was defined as sharing a bed with the infant for at least three nights per week.

### 2.4. Milk Sample Collection

Each participant provided 20 mL of breast milk. Milk was collected at least two hours after the last feeding, from the breast normally used for nursing, to minimize variability. Samples were collected under standardized conditions in a private lactation room using an electric breast pump (Symphony, Medela, Baar, Switzerland). Participants received oral instructions on hygiene procedures. Milk was collected into sterile, disposable 20 mL containers. Samples were transported to the laboratory immediately after expression in isothermal bags. The laboratory was located within the same medical facility, and the transport time did not exceed 3 min. Upon arrival, the samples were processed without delay for flow cytometry. Only fresh, non-frozen milk was used for analysis. Any deviation from this protocol—such as delayed transport or improper handling—resulted in the exclusion of the sample from cytometric analysis.

The following cell populations were evaluated: total leukocytes (CD45^+^); B cells (CD19^+^); NK cells (CD16/56^+^); T cells (CD3^+^) and their subtypes (CD4^+^ helper and CD8^+^ cytotoxic T cells); hematopoietic stem cells (CD45^−^CD105^+^CD34^+^CD326^−^); mesenchymal stem cells (CD45^−^CD105^+^CD73^+^CD44^+^); and lactocytes with phenotypes CD45^−^CD105^+^CD326^+^CD73^+^ and CD45^−^CD105^+^CD326^+^CD73^−^.

### 2.5. Flow Cytometry

A proprietary methodology was developed based on prior experience and adapted specifically for milk analysis.

In this study, 20 mL of cold breast milk and 20 mL of cold 1X Phosphate-Buffered Saline (PBS) were added to a Falcon tube and centrifuged at 1000× *g* Relative Centrifugal Force (RCF) for 20 min at 4 °C. The pellets were rinsed and centrifuged again twice. Pellets from the final centrifugation were resuspended in 1X PBS. Furthermore, 100 µL samples were dispensed into phenotype and stem cell panels and then incubated for 30 min in the dark at room temperature. After staining the samples with antibodies, cells were fixed with 4% paraformaldehyde (PFA) and washed with 1X PBS. Samples labeled with all antibody panels were resuspended in 1X PBS. Stained samples were assayed on a Cytoflex flow cytometer (Beckman Coulter, Brea, CA, USA) and analyzed using Flow Cytometry Analysis Program (FCAP) Array software (Version 2.3). Data were analyzed in dedicated flow cytometry software CytExpert 2.3.

#### 2.5.1. Gating Strategy—Phenotype

For extracellular staining, Dulbecco’s Phosphate-Buffered Saline (DPBS) 10X (Corning), 4% PFA (Merck, Darmstadt, Germany), and appropriate antibody–fluorochrome tandems (Beckman Coulter) were used. Leukocyte Common Antigen: anti-CD45-KRO was utilized to determine the general phenotype of leukocytes. For the characteristics of individual subpopulations among leukocytes (CD45+ cells), the following antibodies were applied: anti-CD3-FITC, anti-CD8-PC5, and anti-CD4-APC for T lymphocytes; anti-CD19-PC 7 for B lymphocytes; and anti-CD(16+56)-PE for NK cells.

In the phenotypic gating strategy, the first step was to exclude dead cells based on the FSC and SSC parameters. The area in which live cells are concentrated was visible, while dead cells and impurities were rejected. The next step was the selection of CD45+ cells, which allowed the isolation of the leukocyte population and the limitation of the analysis to them only.

**Figure A1:**
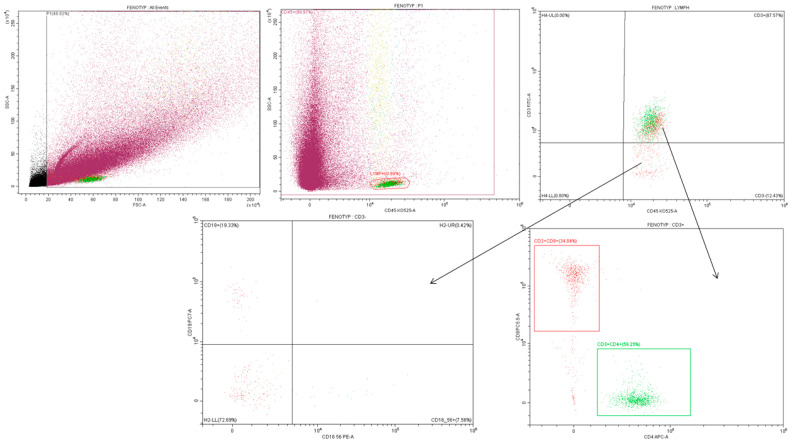
Gating strategy—milk leukocyte phenotype.

In the next steps, the division into lymphocyte subpopulations was performed. CD3+ cells were identified as T lymphocytes, CD19+ as B lymphocytes, and CD(16+56) as NK cells. Additionally, in the T lymphocyte population, the distinction between CD4+ and CD8+ subpopulations was analyzed. CD4+ identified T helper lymphocytes, while CD8+—cytotoxic lymphocytes (Figure A1 in Appendix A).

#### 2.5.2. Gating Strategy—Lactocytes and Stem Cells

In the study material, the cells with the stem cell phenotype were also characterized based on the following markers: CD105-PC7, CD34-ECD, CD73-PE, CD45-AF700, and CD44-AF750. For epithelial cells an anti-CD326-APC antibody was used. Mesenchymal stem cells (MSCs) were characterized using anti-CD45-KRO and anti-CD105-PC7 antibodies. To characterize hematopoietic/precursor stem cells (HSC), anti-CD34-ECD antibodies were used. Additionally, for more precise MSC analysis, anti-CD73-PE antibodies were utilized. For the general characterization of both HSC and MSC, anti-CD44-APC antibodies were applied. Epithelial progenitor cells were characterized using anti-CD326-APC antibodies.

**Figure A2:**
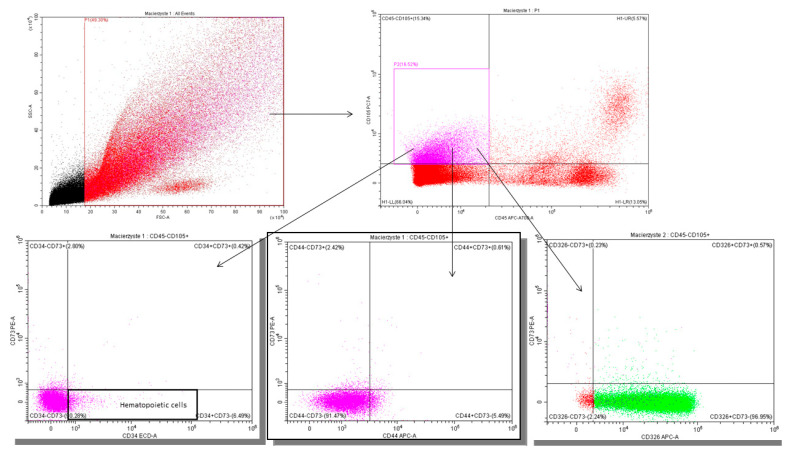
Gating strategy—milk stem cells and epithelial cells.

The stem cell analysis process began with the elimination of dead cells based on FSC (Forward Scatter) and SSC (Side Scatter) parameters. Only living cells were considered in the analysis, while dead cells and impurities were rejected. Then, gating was performed on CD45− cells based on the expression of CD105+. Accordingly, CD45−CD105+ cells were classified as mesenchymal stem cells. In the subsequent stages, gating was carried out based on the expression of specific markers. CD34+ cells are classified as hematopoietic stem cells. The CD44+ and CD73+ subpopulations were identified as mesenchymal stem cells. Cells with the CD326+73+ and CD326+73− phenotypes are classified as lactocytes (Figure A2 in Appendix A).

This study was conducted in accordance with the Declaration of Helsinki and approved by the Bioethics Committee of the Military Medical Chamber (13 May 2022, consent No. 221/22).

### 2.6. Statistical Analysis

No formal sample size calculation was performed due to the exploratory nature of this study. The final sample size was determined by the available recruitment capacity and laboratory throughput during the study period.

The statistical analysis was performed using SPSS Statistics for Windows, version 29.0.0.0 (IBM Corp., New York, NY, USA). In all analyses, *p*-values < 0.05 were considered statistically significant. In the first stage of the analysis, the normality of the distribution of variables was assessed using the Kolmogorov–Smirnov test with Lilliefors correction. For variables with a normal distribution, the mean was used as a central measure and the standard deviation as a measure of dispersion. For variables with a non-normal distribution, the median was used as a central measure and the interquartile range as a measure of dispersion. For comparisons of continuous variables in two groups, the Student’s *t*-test was used for variables with a normal distribution, and the Mann–Whitney U test was used for variables with a non-normal distribution. For comparisons of dichotomous variables, the chi-square test was used, and in the case of its assumptions not being met, the Fisher’s exact test was used. In order to assess the dependence of two continuous variables, correlation calculations were performed, and then multivariate regression analysis was performed. Linear regression models were applied to examine the relationship between immune cell proportions in breast milk and selected domestic factors: number of household members, pet ownership, and bed-sharing. In a second step, multivariate models were adjusted for potential confounding variables, including maternal age, gestational age at delivery, and mode of delivery (vaginal birth vs. cesarean section). Variables were selected based on existing literature and biological plausibility. Stepwise inclusion of covariates served as a sensitivity approach to test the robustness of the observed associations.

## 3. Results

### 3.1. Participant Characteristics

Forty-nine breastfeeding mothers aged 25 to 40 years were included in this study (mean age: 32.0 ± 3.03 years). Their breastfed infants ranged in age from 1 to 6 months (median: 3 months; IQR: 2.0–4.5).

The median gestational age was 39 weeks (IQR: 39.0–40.0). Most participants were multiparous (*n* = 37; 75.5%). Vaginal delivery was the most common mode of birth (*n* = 30; 61%), while cesarean section accounted for 39% (*n* = 19).

Perinatal history was unremarkable for all infants. They were born in good condition (median Apgar score at 5 min: 10; IQR: 10.0–10.0) and were eutrophic (median birth weight: 3650 g; IQR: 3340–3830 g).

Nineteen infants (38.8%) had ever received formula supplementation. Forty infants (82%) were breastfed and held in skin-to-skin contact within the first two hours after birth. Twenty-one infants (43%) had received antibiotic therapy prior to milk sampling.

Environmental conditions varied across households. Co-sleeping was reported by more than half of the mothers (*n* = 25; 51%). Twelve households (24.5%) included pets. Thirty-seven infants (75.5%) lived with at least one sibling. A detailed description of the study group is presented in Table 2.

The distribution of immune and epithelial cell populations in human milk samples from all participants (*N* = 49) is summarized in Table 3.

### 3.2. Impact of Number of Children on Milk Cell Composition

**Figure 1:**
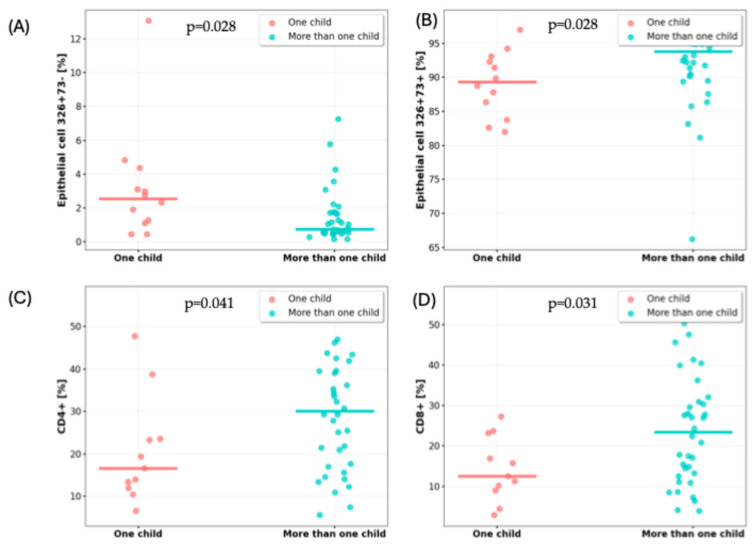
Differences in selected milk cell populations depending on the number of children. (**A**) Proportion of lactocytes with the CD326^+^CD73− phenotype; (**B**) proportion of lactocytes with the CD326^+^CD73^+^ phenotype; (**C**) percentage of CD4^+^ T cells; and (**D**) percentage of CD8^+^ T cells.

The analysis revealed that the number of children was significantly associated with the cellular composition of breast milk. Mothers with one child had a higher percentage of CD326^+^CD73^+^ lactocytes and a lower proportion of CD326^+^CD73^−^ lactocytes compared to mothers with more than one child. In contrast, breast milk from mothers with more than one child contained significantly higher percentages of CD4^+^ helper and CD8^+^ cytotoxic T lymphocytes (Table 4, Figure 1).

There were no statistically significant differences in the proportions of mesenchymal stem cells, hematopoietic progenitor cells, NK cells, or B cells.

Background characteristics were compared between mothers with one child and those with more than one child to assess potential confounders. No significant differences were found in maternal age, gestational age, mode of delivery, infant age, birth weight, or birth length. However, mothers with one child were more likely to initiate breastfeeding within 2 h postpartum (*p* = 0.022) and less likely to supplement with formula milk (*p* = 0.016). To evaluate whether these differences could influence the composition of breast milk, we conducted additional analyses. Neither the timing of breastfeeding initiation nor formula supplementation had a significant impact on cell populations. These findings are summarized in Appendix B Table A1, Table A2 and Table A3.

Increasing household member size was not associated with significant changes in the composition of the analyzed milk cell populations. No statistically significant differences were observed in the proportions of lymphocyte subpopulations, epithelial cells, progenitor cells, or mesenchymal stem cells between mothers with two children and those with three or more.

### 3.3. Impact of Co-Sleeping on Milk Composition

This study demonstrated that co-sleeping was associated with a reduced proportion of T lymphocytes in breast milk. Mothers who practiced co-sleeping had significantly lower levels of CD3^+^ T cells compared to mothers who did not co-sleep with their infants (*p* = 0.021). The percentages of CD4^+^ helper and CD8^+^ cytotoxic T cells were also lower in the co-sleeping group; however, these differences did not reach statistical significance (Table 5).

A multiple linear regression model was used to assess the relationship between the proportion of CD3^+^ T cells in breast milk and selected domestic factors: number of household members, bed-sharing, and pet ownership. The overall model was statistically significant (*p* = 0.029), explaining approximately 18.7% of the variance in CD3^+^ cell proportion (adjusted R^2^ = 0.187). Among the predictors, bed-sharing was significantly associated with a lower percentage of CD3^+^ T cells (β = −0.341, *p* = 0.018). In a subsequent model including maternal and perinatal variables (maternal age, gestational week, birth weight, and delivery mode), only co-sleeping remained a significant predictor of CD3^+^ T cell proportion (*B* = −9.818, *p* = 0.023). Other variables did not show statistically significant associations. The overall model was not significant (*p* = 0.180).

### 3.4. Impact of Pet Ownership on Milk Cell Composition

No significant differences in background characteristics were observed between mothers with and without household pets, including maternal age, gestational age, mode of delivery, infant age, birth weight, birth length, formula supplementation, or timing of breastfeeding initiation.

Data on pet type were available for all participants reporting pet ownership. Among these, six mothers had dogs, five had cats, and one reported both a dog and a cat. Due to the small number of cases in each subgroup, comparative statistical analysis based on pet species was not feasible, and pet ownership was treated as a binary variable in the final analysis.

The presence of animals in the household was significantly associated with the immune profile of human milk. Mothers who reported pet ownership had a significantly lower proportion of CD8^+^ cytotoxic T lymphocytes in their milk compared to mothers without pets (*p* = 0.048). A reduction in CD4^+^ T cells was also observed in the pet-owning group, but this difference did not reach statistical significance (Table 6).

## 4. Discussion

Human milk contains numerous immunological, biochemical, and cellular components that influence neonatal development, immunity, and susceptibility to infections [27].

In addition to cellular factors, human breast milk contains a rich repertoire of bioactive molecules—such as human milk oligosaccharides, microRNAs, and cytokines. These molecules may influence infant health through epigenetic mechanisms. Emerging literature supports that maternal nutrition and milk composition contribute to neonatal immune programming and allergy risk via DNA methylation, histone modifications, and miRNA-mediated gene regulation, particularly during critical early life windows of development [28,29].

The proportions of specific cell types in human milk and the factors affecting them remain poorly understood. This study aimed to evaluate the cellular composition of breast milk in relation to domestic environmental factors. We analyzed the impact of number of household members, number of children, pet ownership, and bed-sharing practices.

Mothers breastfeeding their first child had a higher proportion of CD73^+^ lactocytes and a lower proportion of CD73^−^ lactocytes. Lactocytes are secretory epithelial cells involved in milk synthesis, characterized by cytokeratin 18 and EPCAM (CD326^+^) expression [11]. Initially believed to be apoptotic remnants, later in vitro studies confirmed their viability [30]. In our study, lactocytes were assessed for CD73 surface expression. CD73, or ecto-5′-nucleotidase, is a marker of epithelial activity and plays a role in local inflammatory regulation through adenosine metabolism [31]. The differences in CD73 expression may reflect the gland’s adaptive dynamics between first and subsequent lactations. Higher CD73^+^ levels in mothers with one child may indicate a more active differentiation and remodeling process. In mothers with more than one child, the mammary gland may already display a mature architecture due to previous lactational stimulation. This hypothesis aligns with findings by Russo et al. [32], who demonstrated that both age and parity significantly influence breast gland morphology. Nulliparous breasts predominantly contained undifferentiated structures, whereas multiparous breasts were rich in mature lobules (type 3). Pregnancy induces lasting morphological changes in the mammary gland, potentially offering protection against breast cancer [33,34].

We also observed that the number of children in maternal care influenced milk’s immune profile. Breast milk from mothers with more than one child contained higher levels of CD4^+^ and CD8^+^ T cells. In contrast, the total number of household members had no significant impact. This suggests that differences between mothers with one child and mothers with more than one child were not merely due to household size but to immune experiences and increased microbial exposure inherent to larger families.

Mothers with more than one child may experience more frequent and broader microbial exposure due to the presence of older children. This continuous stimulation of the maternal immune system, particularly through repeated contact with respiratory viruses brought home by siblings, may lead to persistent immune activation. Notably, maternal responses to such exposures do not always manifest with clinical symptoms. Even minimal or subclinical viral contact may induce localized immune responses and inflammation within the mammary gland.

Importantly, although mothers with one child differed in terms of breastfeeding initiation and formula supplementation, these factors did not significantly influence the milk’s cellular composition and thus are unlikely to explain the observed immunological differences.

Recent studies support the view that the mammary gland is immunologically active and responsive to environmental stimuli. Hassiotou et al. showed a rapid and significant leukocyte increase in milk following infant or maternal infection [9]. This response occurred even when only the infant was ill. Similar findings were reported by Zheng et al. [35], who observed elevated T and B cells and increased chemokine levels (CCL20, CXCL10) in maternal milk, independent of maternal symptoms. Riskin et al. [36] also found elevated macrophage counts and TNF-α levels during infant infections. Our previous research confirmed that the mammary immune response to infant infection is multifaceted and influenced by etiology and clinical severity [37].

No previous studies have directly examined the effect of bed-sharing on the immunological composition of milk. However, meta-analyses indicate that bed-sharing increases the frequency of night feedings, enhancing microbial exchange between mother and infant [38]. Only direct breastfeeding enables retrograde flow—where a small amount of infant saliva enters the milk ducts after milk ejection ceases [39]. Saliva–milk interactions generate biochemical metabolites that alter milk composition [40]. Bed-sharing may amplify this process by promoting direct feeding.

Bed-sharing also modulates hormonal axes [41]. Close maternal–infant contact increases oxytocin and stabilizes cortisol secretion [42], both of which influence peripheral tissue activity, including in the mammary gland. This could reduce inflammation and limit CD3^+^ lymphocyte migration into milk. Atanackovic et al. [43] demonstrated that psychological stress alters peripheral T cell profiles, with CD3^+^ cell increases reflecting tissue reservoir mobilization. The lower proportion of CD3^+^ cells found in the milk of mothers who bed-share with their infants, as observed in our study, may be attributed to reduced activation of the neurohormonal stress axis and diminished recruitment of T cells to the mammary gland. This may result in a less inflammatory milk phenotype while maintaining protective functions. The observed CD3^+^ reduction may represent adaptive immune modulation rather than suppression. Further research is needed to confirm this hypothesis.

Interestingly, pet ownership was associated with a lower proportion of CD8^+^ T cells in breast milk. This may reflect a system-wide shift toward immune tolerance. Although studies investigating the relationship between pet ownership and the immunological composition of breast milk are scarce, this finding aligns with literature indicating that domestic environments shape immune development [44,45,46]. Chronic exposure to animal antigens may influence cytokine profiles and reduce the recruitment of effector CD8^+^ T cells to the mammary gland. This supports the “farm effect” hypothesis [47,48], according to which microbially rich environments promote the development of regulatory immune pathways. Langgartner et al. found that animal contact increases FoxP3 expression, which is essential for the differentiation of regulatory T cells [49,50]. By extrapolation, similar mechanisms may be active within the mammary gland. The reduced CD8^+^ cell proportion in pet-exposed mothers may reflect diminished cytotoxic recruitment due to sustained microbial exposure. However, verification of this hypothesis requires studies on cytokine expression and regulatory cell activation in milk.

In summary, this study shows that environmental factors can modulate the cellular composition of human milk. Our findings can be grouped into three main clusters. First, the number of children appears to stimulate immune activity, increasing CD4^+^ and CD8^+^ T cell levels. Second, bed-sharing may enhance microbial harmonization and influence CD3^+^ cell proportions. Third, pet ownership may promote immune tolerance, reflected in reduced CD8^+^ T cell content.

These shifts in the immunological landscape of breast milk may have important consequences for infant health. T lymphocytes transferred via milk can survive passage through the infant gastrointestinal tract and contribute to mucosal immunity, potentially influencing immune maturation and the risk of immune-mediated conditions. Furthermore, the presence of tolerogenic or activated cell populations may modulate local and systemic immune responses in the infant. Although this study did not assess downstream clinical outcomes, the observed differences in cellular composition highlight the possibility that maternal environment indirectly shapes early-life immunity. Long-term studies are needed to evaluate whether these immune signatures in breast milk translate into differences in disease susceptibility or immunological development.

Despite these findings, some limitations must be considered. First, this study focused on cellular composition without microbiome analysis. Second, no functional assessment of isolated cells was performed—activation markers and cytokine profiles were not evaluated. Although such analyses could provide deeper insight into the immunological function of milk leukocytes, they were not feasible due to technical and financial constraints. In addition, advanced techniques such as single-cell transcriptomics, which enable high-resolution analysis of immune cell function and heterogeneity, were beyond the scope of this exploratory study. Third, the sample size was limited. Although 49 samples represent one of the largest cytometric analyses of breast milk in this context, it remains relatively small, particularly for multivariate analysis. Moreover, although we collected data on the species of household pets, the small sample sizes in each subgroup (dogs, cats, or both) limited the possibility of species-specific comparisons. As a result, pet ownership was analyzed as a binary variable. These limitations stem from the exploratory nature of this study. To date, no publications have directly investigated the influence of the domestic environment on milk composition. Our results therefore outline directions for future research.

The findings of this study should be interpreted with caution. Due to its exploratory nature and limited sample size, the observed associations between domestic factors and immune cell composition in breast milk may not reflect causal relationships. Therefore, further research on larger and more diverse populations is needed to confirm these preliminary observations and to explore underlying mechanisms in greater depth.

## 5. Conclusions

In conclusion, domestic factors such as number of children, co-sleeping, and pet exposure are associated with shifts in the immune and lactocyte cell composition of breast milk.

The immunological profile of human milk is not solely shaped by physiological factors. It adapts in response to the environmental context in which the mother–infant dyad functions. These findings support the concept of human milk as a dynamic medium of immune transfer, shaped by complex interactions between domestic microenvironments and mammary gland biology.

## Figures and Tables

**Table 1:** Inclusion and exclusion criteria.

Inclusion Criteria	Exclusion Criteria
Exclusive or predominant breastfeeding	Introduction of solid foods
Infant age between 1 and 6 months	Active respiratory infection (mother or child)
Gestational age ≥ 38 weeks	Chronic illness in mother or infant
Birth weight between the 10th and 90th percentiles	Preterm birth
Written informed consent	History of mastitis
	Lack of informed consent

**Table 2:** Characteristics of the study population.

Variable	Value
Maternal characteristics	
Age [years]; mean (±SD)	32.0 (3.03)
Gestational age [week]; median (IQR)	39.0 (39.0–40.0)
Mode of delivery; *n* (%)	
Vaginal	30 (61)
Cesarean section	19 (39)
Infant characteristics	
Age [months]; median (IQR)	3.22 (1.67)
Sex; *n* (%)	
Female	29 (59)
Male	20 (41)
Birth weight [g]; median (IQR)	3650 (3340–3830)
Birth length [cm]; median (IQR)	55.0 (54.0–57.0)
Apgar score at 5 min [points]; *n* (%)	
10	46 (96)
<10	2 (4)
Additional feeding with formula milk; *n* (%)	19 (39)
Breastfeeding initiated <2 h postpartum; *n* (%)	40 (82)
History of antibiotic therapy; *n* (%)	21 (43)
Environmental conditions	
Siblings; *n* (%)	37 (75.5)
Co-sleeping; *n* (%)	25 (51.0)
Pets in the household; *n* (%)	12 (24.5)
Number of household members; *n* (%)	
3	11 (22.4)
4	30 (61.2)
≥5	8 (16.3)

*n*—number; SD—standard deviation; IQR—interquartile range.

**Table 3:** Distribution of immune and epithelial cell populations in human milk samples from all participants (*N* = 49).

Variable	Value
CD45-CD105+CD34+ [%], median (IQR)	0.44 (0.20–0.98)
CD45-CD105+73+44+ [%], median (IQR)	1.70 (0.84–2.46)
CD45-CD105+326+73+ [%], median (IQR)	1.11 (0.55–2.43)
CD45-CD105+326+73− [%], median (IQR)	92.34 (88.51–95.69)
CD45+ [%], median (IQR)	5.18 (3.39–8.29)
CD3+ [%], median (IQR)	82.43 (70.57–88.81)
CD4+ [%], median (IQR)	25.53 (14.53–39.45)
CD8+ [%], median (IQR)	17.78 (11.06–29.52)
CD16/56+ [%], median (IQR)	6.84 (3.81–11.34)
CD19+ [%], median (IQR)	4.58 (2.49–7.02)

IQR—interquartile range.

**Table 4:** Percentage of immune, epithelial, mesenchymal, and progenitor cells in breast milk from mothers with one child versus more than one child.

Variable	One Child (*n* = 12)	More Than One Child (*n* = 37)	*p*-Value
CD45-CD105+CD34+ [%], median (IQR)	0.77 (0.26–1.17)	0.35 (0.18–0.86)	p^UMW^ = 0.197
CD45-CD105+73+44+ [%], median (IQR)	1.68 (0.84–2.36)	1.73 (0.75–2.46)	p^UMW^ = 0.891
CD45-CD105+326+73+ [%], median (IQR)	2.54 (1.14–4.04)	0.73 (0.53–1.71)	**p^UMW^ = 0.028**
CD45-CD105+326+73− [%], median (IQR)	89.29 (84.38–92.85)	93.79 (89.96–96.23)	**p^UMW^ = 0.028**
CD45+ [%], median (IQR)	5.34 (5.06–8.24)	4.44 (3.00–8.90)	p^UMW^ = 0.449
CD3+ [%], median (IQR)	75.57 (66.24–83.75)	85.46 (71.04–90.60)	p^UMW^ = 0.165
CD4+ [%], mean (±SD)	20.51 (12.53)	29.97 (13.93)	**p^st^ = 0.047**
CD8+ [%], mean (±SD)	14.26 (7.93)	24.13 (13.98)	**p^St^ = 0.031**
CD16/56+ [%], median (IQR)	6.33 (2.06–13.04)	6.85 (4.02–11.07)	p^UMW^ = 0.911
CD19+ [%], median (IQR)	6.60 (3.15–17.40)	4.28 (2.27–5.84)	p^UMW^ = 0.173

*n*—number; SD—standard deviation; IQR—interquartile range; St—Student’s *t*-test; UMW—Mann–Whitney U test. Statistically significant results are shown in bold.

**Table 5:** Cell populations in breast milk from mothers who co-slept with their infants vs. those who did not.

Variable	Co-Sleeping (+) (*n* = 25)	Co-Sleeping (−) (*n* = 24)	*p*-Value
CD45-CD105+CD34+ [%], median (IQR)	0.55 (0.20–1.13)	0.35 (0.20–0.82)	p^UMW^ = 0.317
CD45-CD105+73+44+ [%], median (IQR)	1.85 (0.95–3.57)	1.52 (0.50–1.99)	p^UMW^ = 0.062
CD45-CD105+326+73+ [%], median (IQR)	0.75 (0.54–2.98)	1.12 (0.60–2.32)	p^UMW^ = 0.904
CD45-CD105+326+73− [%], median (IQR)	90.13 (87.53–93.19)	94.85 (91.32–96.26)	p^UMW^ = 0.055
CD45+ [%], median (IQR)	5.18 (3.48–8.82)	5.16 (3.14–6.96)	p^UMW^ = 0.639
CD3+ [%], median (IQR)	75.25 (62.52–86.74)	84.99 (79.41–91.85)	p^UMW^ = 0.021
CD4+ [%], mean (±SD)	27.28 (14.04)	28.17 (14.38)	p^st^ = 0.833
CD8+ [%], mean (±SD)	19.46 (11.20)	23.88 (15.04)	p^St^ = 0.264
CD16/56+ [%], median (IQR)	6.87 (4.43–13.47)	6.25 (3.75–9.93)	p^UMW^ = 0.482
CD19+ [%], median (IQR)	4.81 (2.30–7.00)	4.51 (2.43–7.05)	p^UMW^ = 0.932

*n*—number; SD—standard deviation; IQR—interquartile range; St—Student’s *t*-test; UMW—Mann–Whitney U test.

**Table 6:** Percentage of immune, epithelial, mesenchymal, and progenitor cells in breast milk of mothers with and without pet exposure.

Variable	Pets (+) (*n* = 12)	Pets (−) (*n* = 37)	*p*-Value
CD45-CD105+CD34+ [%], median (IQR)	0.20 (0.12–0.80)	0.47 (0.29–1.02)	p^UMW^ = 0.127
CD45-CD105+73+44+ [%], median (IQR)	1.64 (0.66–2.36)	1.73 (0.81–2.46)	p^UMW^ = 0.608
CD45-CD105+326+73+ [%], median (IQR)	1.07 (0.62–3.95)	1.13 (0.53–2.11)	p^UMW^ = 0.453
CD45-CD105+326+73− [%], median (IQR)	93.62 (86.61–97.01)	92.23 (89.21–95.41)	p^UMW^ = 0.881
CD45+ [%], median (IQR)	5.18 (2.39–8.98)	5.15 (3.96–8.18)	p^UMW^ = 0.931
CD3+ [%], median (IQR)	76.99 (61.84–84.08)	84.55 (72.91–89.50)	p^UMW^ = 0.378
CD4+ [%], mean (±SD)	1.98 (14.00)	29.52 (13.81)	p^st^ = 0.121
CD8+ [%], median (IQR)	14.39 (7.22–17.11)	23.73 (12.50–30.70)	p^UMW^ = 0.048
CD16/56+ [%], median (IQR)	9.22 (1.59–11.34)	6.39 (4.71–11.10)	p^UMW^ = 0.951
CD19+ [%], median (IQR)	4.58 (2.81–7.19)	4.59 (2.23–6.97)	p^UMW^ = 0.701

*n*—number; SD—standard deviation; IQR—interquartile range; St—Student’s *t*-test; UMW—Mann–Whitney U test.

## Data Availability

The datasets used and/or analyzed during the current study are available from the corresponding author upon reasonable request. The datasets are not publicly available due to privacy reason.

## Appendix B

**Table A1:** Comparison of background maternal and infant characteristics between mothers with one child and mothers with more than one child.

Variable	One Child (*n* = 12)	More Than One Child (*n* = 37)	*p*-Value
Maternal characteristics			
Age [years]; mean (±SD)	30.92 (2.81)	32.65 (3.01)	p^St^ = 0.085
Gestational age [week]; median (IQR)	40.0 (39.0–40.0)	39.0 (39.0–40.0)	p^UMW^ = 0.415
Mode of delivery; *n* (%)			
Vaginal	5 (41.7)	25 (67.6)	P^chi2^ = 0.110
Cesarean section	7 (58.3)	12 (32.4)	
Infant characteristics			
Age [months]; mean (±SD)	3.83 (1.46)	3.03 (1.70)	p^UMW^ = 0.149
Birth weight [g]; median (IQR)	3765 (3340–3995)	3600 (3340–3800)	p^UMW^ = 0.396
Birth length [cm]; median (IQR)	55.0 (53.25–58.75)	55.0 (54.0–56.50)	p^UMW^ = 0.589
Additional feeding with formula milk; *n* (%)	8 (66.7)	11 (29.7)	**P^chi2^ = 0.022**
Breastfeeding initiated <2 h postpartum; *n* (%)	7 (58.3)	33 (89.2)	**P^chi2^ = 0.016**

*n*—number; SD—standard deviation; IQR—interquartile range; St—Student’s *t*-test; UMW—Mann–Whitney U test; chi2—chi-square test (asymptotic, two-sided). Statistically significant results are shown in bold.

**Table A2:** Comparison of cell populations in breast milk according to formula supplementation.

Variable	Extra Feeding with Formula Milk (*n* = 19)	No Extra Feeding with Formula Milk (*n* = 30)	*p*-Value
CD45-CD105+CD34+ [%], median (IQR)	0.62 (0.18–0.97)	0.39 (0.21–1.19)	p^UMW^ = 0.901
CD45-CD105+73+44+ [%], median (IQR)	1.73 (0.56–1.91)	1.68 (0.83–2.91)	p^UMW^ = 0.605
CD45-CD105+326+73+ [%], median (IQR)	1.83 (0.97–3.21)	0.70 (0.49–1.70)	p^UMW^ = 0.051
CD45-CD105+326+73− [%], median (IQR)	91.35 (86.15–95.69)	93.02 (89.56–95.92)	p^UMW^ = 0.333
CD45+ [%], median (IQR)	5.16 (4.10–6.49)	5.18 (2.82–14.03)	p^UMW^ = 0.562
CD3+ [%], median (IQR)	83.09 (75.24–92.03)	80.58 (62.52–88.79)	p^UMW^ = 0.431
CD4+ [%], mean (±SD)	27.80 (12.15)	27.72 (15.36)	p^st^ = 0.493
CD8+ [%], mean (±SD)	22.39 (12.58)	21.45 (14.13)	p^St^ = 0.410
CD16/56+ [%], median (IQR)	5.82 (3.18–9.82)	7.64 (4.88–12.73)	p^UMW^ = 0.237
CD19+ [%], median (IQR)	3.81 (1.71–8.21)	4.82 (2.52–7.09)	p^UMW^ = 0.406

*n*—number; SD—standard deviation; IQR—interquartile range; St—Student’s *t*-test; UMW—Mann–Whitney U test.

**Table A3:** Comparison of composition in breast milk according to time of breastfeeding initiation.

Variable	Breastfeeding Initiated > 2 h (*n* = 9)	Breastfeeding Initiated < 2 h (*n* = 40)	*p*-Value
CD45-CD105+CD34+ [%], median (IQR)	0.63 (0.15–1.42)	0.45 (0.21–0.99)	p^UMW^ = 0.805
CD45-CD105+73+44+ [%], median (IQR)	2.11 (0.67–4.92)	1.68 (0.81–2.01)	p^UMW^ = 0.505
CD45-CD105+326+73+ [%], median (IQR)	1.25 (0.47–4.01)	1.10 (0.55–2.24)	p^UMW^ = 0.919
CD45-CD105+326+73− [%], median (IQR)	90.75 (86.66–95.10)	93.02 (89.21–95.73)	p^UMW^ = 0.354
CD45+ [%], median (IQR)	5.34 (4.04–18.14)	4.80 (3.29–7.95)	p^UMW^ = 0.317
CD3+ [%], median (IQR)	83.75 (63.44–93.66)	81.50 (69.55–88.64)	p^UMW^ = 0.449
CD4+ [%], mean (±SD)	28.00 (16.50)	27.69 (13.69)	p^st^ = 0.954
CD8+ [%], mean (±SD)	20.72 (13.56)	22.08 (13.56)	p^St^ = 0.788
CD16/56+ [%], median (IQR)	6.33 (2.93–8.76)	7.20 (4.39–13.16)	p^UMW^ = 0.344
CD19+ [%], median (IQR)	4.82 (1.49–22.51)	4.51 (2.55–6.87)	p^UMW^ = 0.871

*n*—number; SD—standard deviation; IQR—interquartile range; St—Student’s *t*-test; UMW—Mann–Whitney U test.

## References

[1] Carr L.E., Virmani M.D., Rosa F., Munblit D., Matazel K.S., Elolimy A.A., Yeruva L. (2021). Role of Human Milk Bioactives on Infants’ Gut and Immune Health. Front. Immunol..

[2] Szyller H., Antosz K., Batko J., Mytych A., Dziedziak M., Wrześniewska M., Braksator J., Pytrus T. (2024). Bioactive Components of Human Milk and Their Impact on Child’s Health and Development, Literature Review. Nutrients.

[3] Eisha S., Joarder I., Wijenayake S., McGowan P.O. (2022). Non-nutritive bioactive components in maternal milk and offspring development: A scoping review. J. Dev. Orig. Health Dis..

[4] Gregg B., Ellsworth L., Pavela G., Shah K., Berger P.K., Isganaitis E., VanOmen S., Demerath E.W., Fields D.A. (2022). Bioactive compounds in mothers milk affecting offspring outcomes: A narrative review. Pediatr. Obes..

[5] Slater A.S., Hickey R.M., Davey G.P. (2025). Interactions of human milk oligosaccharides with the immune system. Front. Immunol..

[6] Peroni D.G., Chirumbolo S., Veneri D., Piacentini G.L., Tenero L., Vella A., Ortolani R., Raffaelli R., Boner A.L. (2013). Colostrum derived B and T cells as an extra lymphoid compartment of effector cell populations in humans. J. Matern.-Fetal Neonatal Med..

[7] Patki S., Kadam S., Chandra V., Bhonde R. (2010). Human breast milk is a rich source of multipotent mesenchymal stem cells. Hum. Cell.

[8] Kaingade P.M., Somasundaram I., Nikam A.B., Sarang S.A., Patel J.S. (2016). Assessment of growth factors secreted by human breastmilk mesenchymal stem cells. Breastfeed. Med..

[9] Hassiotou F., Geddes D.T. (2015). Immune cell mediated protection of the mammary gland and the infant during breastfeeding. Adv. Nutr..

[10] Fan Y., Chong Y.S., Choolani M.A., Cregan M.D., Chan J.K.Y. (2010). Unravelling the mystery of stem/progenitor cells in human breast milk. PLoS ONE.

[11] Witkowska Zimny M., Kaminska El Hassan E. (2017). Cells of human breast milk. Cell. Mol. Biol. Lett..

[12] Twigger A.J., Hepworth A.R., Tat Lai C., Chetwynd E., Stuebe A.M., Blancafort P., Hartmann P.E., Geddes D.T., Kakulas F. (2015). Gene expression in breastmilk cells is associated with maternal and infant characteristics. Sci. Rep..

[13] Field C.J. (2005). The immunological components of human milk and their effect on immune development in infants. J. Nutr..

[14] Ninkina N., Kukharsky M.S., Hewitt M.V., Lysikova E.A., Skuratovska L.N., Deykin A.V., Buchman V.L. (2019). Stem cells in human breast milk. Hum. Cell.

[15] Briere C.E., Jensen T., McGrath J.M., Young E.E., Finck C. (2017). Stem like cell characteristics from breast milk of mothers with preterm infants as compared to mothers with term infants. Breastfeed. Med..

[16] Enstad S., Cheema S., Thomas R., Fichorova R.N., Martin C.R., O’Tierney Ginn P., Wagner C.L., Sen S. (2021). The impact of maternal obesity and breast milk inflammation on developmental programming of infant growth. Eur. J. Clin. Nutr..

[17] Du Y., Yang M., Lee S., Behrendt C.L., Hooper L.V., Saghatelian A., Wan Y. (2012). Maternal Western diet causes inflammatory milk and TLR2/4 dependent neonatal toxicity. Genes Dev..

[18] Whitaker K.M., Marino R.C., Haapala J.L., Foster L., Smith K.D., Teague A.M., Jacobs D.R., Fontaine P.L., McGovern P.M., Schoenfuss T.C. (2017). Associations of maternal weight status before, during, and after pregnancy with inflammatory markers in breast milk. Obesity.

[19] Panagos P.G., Vishwanathan R., Penfield Cyr A., Matthan N.R., Shivappa N., Wirth M.D., Hebert J.R., Sen S. (2016). Breastmilk from obese mothers has pro inflammatory properties and decreased neuroprotective factors. J. Perinatol..

[20] Sharp J.A., Lefèvre C., Watt A., Nicholas K.R. (2016). Analysis of human breast milk cells: Gene expression profiles during pregnancy, lactation, involution, and mastitic infection. Funct. Integr. Genom..

[21] Rio Aige K., Azagra Boronat I., Castell M., Selma Royo M., Collado M.C., Rodríguez Lagunas M.J., Pérez Cano F.J. (2021). The breast milk immunoglobulinome. Nutrients.

[22] Arenas G., Barrera M.J., Contreras Duarte S. (2025). The impact of maternal chronic inflammatory conditions on breast milk composition: Possible influence on offspring metabolic programming. Nutrients.

[23] Sitarik A.R., Havstad S., Levin A.M., Lynch S.V., Fujimura K.E., Ownby D.R., Johnson C.C., Wegienka G. (2018). Dog introduction alters the home dust microbiota. Indoor Air.

[24] Baddock S.A., Purnell M.T., Blair P.S., Pease A., Elder D., Galland B.C. (2019). The influence of bed sharing on infant physiology, breastfeeding and behaviour: A systematic review. Sleep Med. Rev..

[25] Christensen E.D., Hjelmsø M.H., Thorsen J., Shah S., Redgwell T., Poulsen C.E., Trivedi U., Russel J., Gupta S., Chawes B.L. (2022). The developing airway and gut microbiota in early life is influenced by age of older siblings. Microbiome.

[26] Bianco I., Ferrara C., Romano F., Loperfido F., Sottotetti F., El Masri D., Vincenti A., Cena H., De Giuseppe R. (2024). The influence of maternal lifestyle factors on human breast milk microbial composition: A narrative review. Biomedicines.

[27] Yi D.Y., Kim S.Y. (2021). Human breast milk composition and function in human health: From nutritional components to microbiome and microRNAs. Nutrients.

[28] van Esch B.C.A.M., Porbahaie M., Abbring S., Garssen J., Potaczek D.P., Savelkoul H.F.J., van Neerven R.J.J. (2020). The Impact of Milk and Its Components on Epigenetic Programming of Immune Function in Early Life and Beyond: Implications for Allergy and Asthma. Front. Immunol..

[29] Acevedo N., Alashkar Alhamwe B., Caraballo L., Ding M., Ferrante A., Garn H., Garssen J., Hii C.S., Irvine J., Llinás-Caballero K. (2021). Perinatal and Early-Life Nutrition, Epigenetics, and Allergy. Nutrients.

[30] Alsaweed M., Hepworth A.R., Lefèvre C., Hartmann P.E., Geddes D.T., Hassiotou F. (2015). Human milk microRNA and total RNA differ depending on milk fractionation. J. Cell. Biochem..

[31] Colgan S.P., Eltzschig H.K., Eckle T., Thompson L.F. (2006). Physiological roles for ecto-5′-nucleotidase (CD73). Purinergic Signal..

[32] Russo J., Rivera R., Russo I.H. (1992). Influence of age and parity on the development of the human breast. Breast Cancer Res. Treat..

[33] Surdacka L.M., Jakubas A., Jagiełło J., Daniłowska K., Picheta N., Gil Kulik P. (2024). Epigenetic and immune mechanisms linking breastfeeding to lower breast cancer rates. Med. Sci. Monit..

[34] Slepicka P.F., Somasundara A.V.H., dos Santos C.O. (2021). The molecular basis of mammary gland development and epithelial differentiation. Semin. Cell Dev. Biol..

[35] Zheng Y., Corrêa Silva S., Rodrigues R.M., Corrêa de Souza E., Macaferri da Fonseca F.A., Gilio A.E., Carneiro Sampaio M., Palmeira P. (2024). Infant respiratory infections modulate lymphocyte homing to breast milk. Front. Immunol..

[36] Riskin A., Almog M., Peri R., Halasz K., Srugo I., Kessel A. (2012). Changes in immunomodulatory constituents of human milk in response to active infection in the nursing infant. Pediatr. Res..

[37] Tomaszewska A., Jeleniewska A., Porębska K., Królikowska K., Rustecka A., Lipińska Opałka A., Będzichowska A., Zdanowski R., Aleksandrowicz K., Kloc M. (2023). Immunomodulatory effect of infectious disease of a breastfed child on the cellular composition of breast milk. Nutrients.

[38] Wolf R.L., Skobic I., Pope B.T., Zhu A., Chamas H., Sharma N., Larsen K.M., Bright H.S., Haynes P.L. (2025). Mother infant bed sharing is associated with increased breastfeeding: A systematic review. Breastfeed. Med..

[39] Beghetti I., Biagi E., Martini S., Brigidi P., Corvaglia L., Aceti A. (2019). Human milk’s hidden gift: Implications of the milk microbiome for preterm infants’ health. Nutrients.

[40] Al Shehri S.S., Knox C.L., Liley H.G., Cowley D.M., Wright J.R., Henman M.G., Hewavitharana A.K., Charles B.G., Shaw P.N., Sweeney E.L. (2015). Breastmilk saliva interactions boost innate immunity by regulating the oral microbiome in early infancy. PLoS ONE.

[41] Matyas M., Apanasewicz A., Krzystek Korpacka M., Jamrozik N., Cierniak A., Babiszewska Aksamit M., Ziomkiewicz A. (2024). The association between maternal stress and human milk concentrations of cortisol and prolactin. Sci. Rep..

[42] Simon C.D., Adam E.K., McKinney C.O., Krohn J.B., Shalowitz M.U. (2016). Breastfeeding, bed sharing, and maternal cortisol. Clin. Pediatr..

[43] Atanackovic D., Nowottne U., Freier E., Weber C.S., Meyer S., Bartels K., Hildebrandt Y., Cao Y., Kröger N., Brunner Weinzierl M.C. (2013). Acute psychological stress increases peripheral blood CD3^+^CD56^+^ natural killer T cells in healthy men: Possible implications for the development and treatment of allergic and autoimmune disorders. Stress.

[44] Abdolghanizadeh S., Salmeh E., Mirzakhani F., Soroush E., Siadat S.D., Tarashi S. (2024). Microbiota insights into pet ownership and human health. Res. Vet. Sci..

[45] Nielsen C.C., Gascon M., Osornio Vargas A.R., Shier C., Guttman D.S., Becker A.B., Azad M.B., Sears M.R., Lefebvre D.L., Moraes T.J. (2020). Natural environments in the urban context and gut microbiota in infants. Environ. Int..

[46] Gensollen T., Iyer S.S., Kasper D.L., Blumberg R.S. (2016). How colonization by microbiota in early life shapes the immune system. Science.

[47] Lee Sarwar K. (2021). The farm like effect: Rural exposures in early life, the microbiome, and asthma. J. Allergy Clin. Immunol..

[48] Kirjavainen P.V., Karvonen A.M., Adams R.I., Täubel M., Roponen M., Tuoresmäki P., Loss G., Jayaprakash B., Depner M., Ege M.J. (2019). Farm like indoor microbiota in non farm homes protects children from asthma development. Nat. Med..

[49] Langgartner D., Weimer K., Brunner Weisser J., Winkler R., Mannes M., Huber Lang M., Sterrett J.D., Lowry C.A., Rohleder N., Bajrami B. (2025). Pawsitive impact: How pet contact ameliorates adult inflammatory stress responses in individuals raised in an urban environment. Brain Behav. Immun..

[50] Fontenot J.D., Gavin M.A., Rudensky A.Y. (2017). Foxp3 programs the development and function of CD4^+^CD25^+^ regulatory T cells. J. Immunol..

